# Comparative Analysis of High-Throughput Assays of Family-1 Plant Glycosyltransferases

**DOI:** 10.3390/ijms21062208

**Published:** 2020-03-23

**Authors:** Kate McGraphery, Wilfried Schwab

**Affiliations:** Biotechnology of Natural Products, Technische Universität München, 85354 Freising, Germany; kate.mcgraphery@tum.de

**Keywords:** glycosyltransferase, high-throughput assay, family-1 UGTs, UDP-Glo assay, phosphate GT assay, screening, kinetics

## Abstract

The ability of glycosyltransferases (GTs) to reduce volatility, increase solubility, and thus alter the bioavailability of small molecules through glycosylation has attracted immense attention in pharmaceutical, nutraceutical, and cosmeceutical industries. The lack of GTs known and the scarcity of high-throughput (HTP) available methods, hinders the extrapolation of further novel applications. In this study, the applicability of new GT-assays suitable for HTP screening was tested and compared with regard to harmlessness, robustness, cost-effectiveness and reproducibility. The UDP-Glo GT-assay, Phosphate GT Activity assay, pH-sensitive GT-assay, and UDP^2^-TR-FRET assay were applied and tailored to plant UDP GTs (UGTs). *Vitis vinifera* (UGT72B27) GT was subjected to glycosylation reaction with various phenolics. Substrate screening and kinetic parameters were evaluated. The pH-sensitive assay and the UDP^2^-TR-FRET assay were incomparable and unsuitable for HTP plant GT-1 family UGT screening. Furthermore, the UDP-Glo GT-assay and the Phosphate GT Activity assay yielded closely similar and reproducible K_M_, v_max_, and k_cat_ values. Therefore, with the easy experimental set-up and rapid readout, the two assays are suitable for HTP screening and quantitative kinetic analysis of plant UGTs. This research sheds light on new and emerging HTP assays, which will allow for analysis of novel family-1 plant GTs and will uncover further applications.

## 1. Introduction

The vast molecular diversity and complexity of various oligosaccharide structures and secondary metabolites across all domains of life [1] result from a coherent interplay of enzymatic reactions encompassing the formation and breakdown of glycosidic linkages [2]. Enzymes such as glycosyltransferases (GTs) play a crucial role in the enzymatic and chemoenzymatic synthesis of oligosaccharides and glycoconjugates [3]. They catalyze the formation of a glycosidic bond by transferring a sugar moiety from an activated glycosyl donor to a suitable acceptor substrate [2]. Their role in many biological and natural processes has attracted a lot of attention over the last few decades [4,5,6,7,8].

The glycosylation of proteins, saccharides, lipids, and small molecules within different organisms involves hundreds of diverse GTs. Being an unusually large enzyme family (with more than 106 sub-families) GTs can be classified within different sub-families depending on their structural and functional similarities [9,10,11]. Furthermore, the GT families are grouped hierarchically from their 3D structures (fold GT-A, GT-B, GT-C, and the proposed GT-D) [12] to their mechanism of reaction (inverting or retaining GTs) [10,13]. Currently, the universally accepted classification of the GT families is mainly established upon sequence similarity collected in the Carbohydrate Active Enzyme database (CAZy, http://www.cazy.org) [14]. The nucleotide-sugar (glycosyl donor) dependent GTs belong to the Leloir enzymes and the glycosyl transfer often occurs at the nucleophilic oxygen of a hydroxyl substituent of the acceptor [2]. GT family-1 of the CAZy classification, also referred to as UDP GTs (UGTs), are the largest GT family in plants that catalyze the transfer of a glycosyl moiety from UDP sugars to a wide range of small molecule acceptors [13]. Crystal structures from various plant UGTs have been analyzed and despite relatively low sequence identities, they all possess the GT-B fold consisting of two Rossmann domains – β/α/β [2,11,15,16,17,18]. The majority of GT-A fold enzymes require divalent cations such as Mg^2+^ and Mn^2+^ for enzymatic activity and contain a DxD motif involved in the binding of the metal. However, GT-B fold enzymes, including plant UGTs of GT family-1, do not appear to require divalent cations for activity and accordingly lack the DxD motif [11]. Plant UGTs are inverting GTs, which employ a direct displacement S_N_2-like reaction. UGTs encompass a vital role in stabilization, enhancement of water solubility and deactivation/detoxification of natural products, leading to regulation of metabolic homeostasis, detoxification of xenobiotics, and the biosynthesis, storage and transportation properties of secondary metabolites [13]. Present across all domains of life, in plants, UGTs are commonly localized in the cytosol playing a vital role in the biosynthesis of secondary metabolites such as flavonoids, phenylpropanoids, terpenoids, steroids, and regulation of plant hormones [19]. Although each GT within this GT family-1 has quite a high sequence divergence, they all possess a consensus sequence at the C-terminal end, which is involved in binding to the UDP moiety of the sugar nucleotide. This consensus sequence comprises 44 amino acid residues and occurs in all GT1 enzymes [20].

Understanding the roles of GTs in biosynthetic pathways is key to understanding various biological processes. Their unique and yet simple mechanism allows for further manipulation and utility across numerous applications [21]. Moreover, the pharmaceutical and nutraceutical properties of small molecules have attracted significant attention from chemical and food industries. The biological functionalities of small molecules may be enhanced by increasing their hydrophilicity and stability through glycosylation [22]. Therefore, screening and detection of glycosylation reactions is crucial in employing further applications. Moreover, screening that is prone to high-throughput involves miniaturization, automation, and rapid readouts of a specific question under study [23]. However, due to the lack of high-throughput and reliable assays available for plant UGTs further and additional applications remain elusive. Therefore, a high-throughput method, which will allow for a rapid and qualitative analysis, is necessary in order to efficiently and effectively screen through a large number of glycosylation reactions.

Some of the current assay methods for GTs were thoroughly reviewed [24]. For example, radiochemical assays are frequently utilized due to their great sensitivity, which allows quantifying even very low concentrations. Separating the non-radiolabeled substrate of the reaction from the radiolabeled product in order to quantify the amount of glycoside achieved can be done by various separation methods, however all are time consuming and not completely quantitative due to often insufficient separation. Moreover, utilizing and disposing of commercially available radiolabeled sugars is expensive and poses health and environmental hazards [25]. Furthermore, immunological methods are also sensitive when it comes to identifying glycosylation products but the requirement of specific antibodies is expensive and often may not be readily available [26]. Enzyme assays and product identification can be combined through different chromatographic methods developed for GT assays. However, usually these methods require substrate fluorescence labeling or particular detection methods [27]. Moreover, mass spectrometry (MS) assays have the advantage of speed and accuracy but are not commonly used as the instrumentation bares extreme high costs [28]. The previous and available methods as well as some recent modifications [29] requiring substrate labeling, high instrument investment, or supplementary enzymes and antibodies, are all based on the detection of the substrate consumption or formation of the nucleotide product [25]. While methods for direct detection of the glycoside products such as, liquid chromatography coupled with mass spectrometry (LC-MS) and use of radiolabeled sugar donors have been established, they remain tedious, expensive, and hazardous [24]. At the same time, the use of LC-MS is unavoidable and is of great valuable importance when analyzing GT reactions to confirm product formation. Moreover, the lack of GTs and/or other suitable sugar donors [30] restricts the alternative application of secondary GTs as reporter enzymes for activity assays of primary GTs [31]. The development of a general 1-Zn(II) NDP sensor assay for rapid evaluation of GT activity was also described [31].

**Figure 1:**
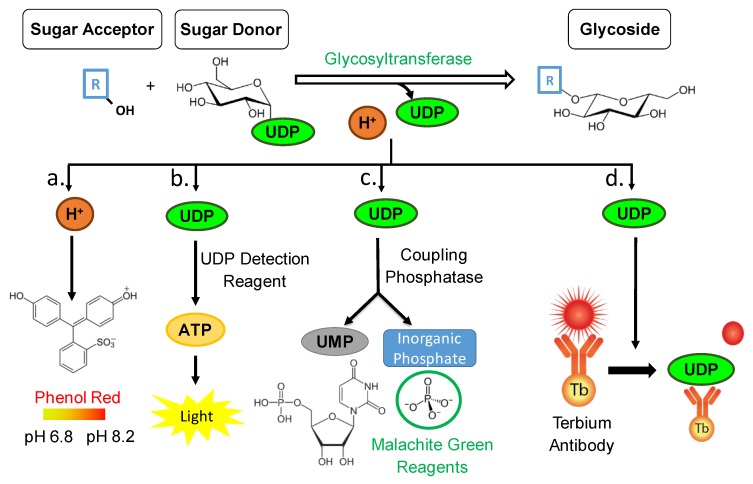
Glycosyltransferase reaction mechanism resulting in the formation of a glycoside. The quantity of by-products is detected by four different assays prone to be high-throughput (HTP). (**a**) Colorimetric pH-sensitive assay—the relationship between the amount of glycoside formed to the pH value is inversely proportional. (**b**) UDP-Glo^TM^ assay—UDP is converted to ATP which triggers a luciferase reaction and generates light. The relationship between the amount of glycoside formed to the amount of ATP detected is directly proportional. (**c**) Phosphate GT assay—a 2-step colorimetric assay utilizing phosphatase and malachite green reagents. The relationship between the amount of glycoside formed to the formation of phosphate is directly proportional. (**d**) UDP^2^ TR-FRET immunoassay—a competitive immunoassay for UDP with a far-red, TR-FRET readout. The relationship between the amount of glycoside formed to the FRET signal is inversely proportional.

Glycosylation catalyzed by UGTs not only results in the formation of a glycosylated product but also of byproducts such as, a proton from the acceptor molecule, and the free UDP molecule from the sugar donor (Figure 1). Therefore, it is viable to quantify not only the glycosylated product formed, but also the amount of byproduct. In the case of the pH-sensitive GT assay the proton byproduct is quantified, and according to the reaction scheme its amount is directly proportional to the amount of glycoside (Figure 1) [25]. The pH change that accompanies the GT-catalyzed reactions can be conveniently used in assay development, and aside from GT reaction can further be used in assays for a variety of other enzymes including kinase [32], lipase [33], and phospholipase [34]. A pH-sensitive assay for mammalian β-1,4-galactosyltransferases (GalT1) to rapidly screen change in pH in a glycosylation reaction has already been described (Table 1) [25]. Glucose, galactose, lactose, *N*-acetylgalactosamine, and glucosamine served as acceptor substrates and UDP-glucose, and UDP-galactose were used as donors. The authors were able to stabilize and develop this method and deemed it sensitive, user friendly and a good advancement in current methodology with regard to initially testing GT functionality. This method was further adapted by us for *Vitis vinifera* GTs to determine kinetic parameters for multiple substrates [35]. However, it was not performed under high-throughput conditions. In this study, we further optimize this assay to establish a high-throughput pH-sensitive GT assay with lower reaction volumes, automated component addition, and fast data acquisition via a multi-microplate instrument and finally compare the obtained results with [35].

Aside from quantifying the hydrogen byproduct from a glycosylation reaction, it is also possible to quantify the amount of UDP released from the sugar donor (Figure 1). By utilizing and adapting the UDP-Glo^TM^ GT Activity Assay from Promega© the UDP amounts are investigated, and according to the reaction scheme the amount of UDP is directly proportional to the amount of formed glycosylated product (Figure 1). This bioluminescent assay is a homogeneous, single/reagent addition method that is able to rapidly detect the formation of UDP in GT reactions. A reagent is added to simultaneously convert the UDP product to ATP and generate light in a luciferase reaction. This assay relies on the properties of a proprietary thermostable luciferase that is formulated to generate a stable glow-type luminescent signal and improve performance across a wide range of assay conditions. The signal produced by the luciferase reaction, which is initiated by adding the ‘UDP Detection Reagent’ which is stable for more than three hours (Figure 1). This extended stability allows for the flexibility of batch-mode processing of multiple plates, simultaneously. This assay has been experimented with various GTs and different substrates (Table 1) [36]. Various applications of the UDP/UMP/GDP-Glo nucleotide detection assays were studied including glycan biosynthesis, post-translational modifications, and drug metabolism [36]. Moreover, the specificity of transfer of different sugars to different acceptors by diverse GTs such as human recombinant GT (ST6GALT1) was analyzed [37] (Table 1). Their findings proved that this bioluminescent platform detects the activity of any nucleotide-sugar using GT regardless of chemical structure, and kinetic parameters could be determined for different sugars [36]. Interestingly, this method has not been employed and experimented in HTP format utilizing plant family-1 GTs. A single instance where this method was proven to be successful and stable in family-1 plant GTs was in one of our recent studies [38].

Moreover, to quantifying the UDP byproduct after conversion to ATP and measuring the luciferase generated light, it is also possible to quantify the phosphate molecules resulting from phosphatase enzymatic cleavage of the UDP (Figure 1). By utilizing and adapting the Phosphate GT Activity assay from R&D Systems© the phosphate amounts are investigated, and according to the reaction scheme the amount of phosphate is directly proportional to the amount of formed glycosylated product. It is a simple, non-radioactive, and high-throughput compatible assay able to determine the enzyme activity of all GTs that use di-phospho-nucleotide sugars as donor substrates. A specific phosphatase is utilized to remove an inorganic phosphate quantitatively from UDP. The released inorganic phosphate is subsequently quantitated by the sensitive colorimetric malachite green phosphate detecting reagents. The amount of inorganic phosphate released by the coupling phosphatase is equal to the nucleotide sugar consumed or glycoconjugate product generated; therefore, the rate of inorganic phosphate products reflects the kinetics of a GT reaction (Figure 1). The phosphatase-coupled GT assay was utilized with various GTs such as, *Clostridium difficile* toxin B, human KTELC1, and human sialyltransferase ST6GAL1 [39] (Table 1).

It is also possible to quantify the amount of UDP released from the sugar donor via a commercially available immunoassay (Figure 1). By utilizing and adapting the Transcreener UDP^2^ TR-FRET Glycosyltransferase Assay from Bellbrook labs© the UDP amounts are quantified. As the free UDP molecules are bound to the antibody, the FRET signal is depleted. It is a competitive immunoassay for UDP with a far-red, time-resolved Förster-resonance-energy-transfer (TR-FRET) readout (Figure 1) and is prone for high-throughput screening with a single addition, mix-and-read format. A similar assay (detecting ADP) was utilized with GmSuSy and PdST GTs [40] and GALNT3 [41] (Table 1).

The application spectrum of GTs and their glycoside products is immense in many aspects of cosmetics, food industry, and drug design [8,42,43]. In order to investigate further innovative applications and find new emerging GTs with unprecedented catalytic activities a robust, high-throughput, and reliable method is sought for. In this study, the colorimetric pH-based assay, two enzyme-coupled assays (UDP-Glo and coupled phosphatase assay), and one immunological assay (Transcreener UDP^2^ TR-FRET) were selected, employed and tailored to suit a plant UGT from *Vitis vinifera*, UGT72B27. These assays have been chosen based on their ease of availability, user-friendliness, advertised robustness, and rapid output. The substrate screening and kinetics were executed with all four chosen methods utilizing one plant GT and various plant secondary metabolites. Furthermore, following the employment of the assays their advantages and disadvantages were analyzed. The aim was to select a high-throughput method that could be utilized for screening of plant family-1 GTs with various substrates (Figure S1).

## 2. Results

All following experimental results were executed with the utilization of a purified UGT72B27 from *Escherichia coli* (Figure S2).

### 2.1. The pH-Sensitive Colorimetric Glycosyltransferase Activity Assay

The assay was employed from [25] and used for the determination of kinetic data of UGT72B27 [35] (Table 2). In this study, the method from [25] was adapted to HTP and the kinetic results [35] were attempted to be reproduced and compared. The experimental setup included a 96-well plate where all the reaction components were added except for the sugar donor (UDP-glucose) and the pH indicator phenol red. The program on the microplate reader was set-up where the injector functions were adding the appropriate amount of UDP-glucose and phenol red in a sequential manner, thereby validating its high-throughput potential. The shaker function and incubation function of the multi-plate reader was employed for the automation of the mixing of the reaction components and incubating the reaction at the appropriate temperature. Following the reaction time, the measurements at the wavelength of 557 nm were obtained. However, the HTP adaptation by utilizing automatic injector function and incubation in a multi-well plate was unsuccessful. The data of the technical and biological replicates varied considerably; reproducible results were unattainable. The pH-sensitive assay appears to be very susceptible to interferences and therefore it was concluded that this colorimetric assay cannot be executed in a high-throughput format in a microplate reader with automatic injector and shaker. In this study, for the comparison of the kinetic properties with subsequent assays the numerical data from [35] was utilized (Table 2).

### 2.2. UDP-Glo^TM^ Glycosyltransferase Activity Assay

The protocol was employed and adapted from the manufacturer whilst tailored to fit family-1 plant GTs. Prior to utilizing this assay, all the metrics and measurement thresholds were identified and tested to ensure it is able to function with plant GTs and hydrophobic substrates. For example, the plate reader’s illuminometer program was adjusted to fit the assay with the appropriate gain and focal length. As well as, the DMSO concentrations were adjusted to ensure proper and unhindered luminescence signals in order to remain within the reagent’s threshold. Finally, the standard curve with increasing amount of UDP was executed. Since we still detected UDP-forming activity of UGT72B27 after addition of the UDR stopping agent we incorporated an additional heat-stop inactivation step at 75 °C after the enzyme reaction to ensure the termination of the catalysis of the plant enzyme. The UDR stopping agent provided by the manufacturer contains a reagent, which terminates the enzymatic reaction of a metal-depending GT (Figure S3) [2]. Although divalent cations (e.g., Mg^2+^) are required for full activity of GT-B enzymes, including plant enzymes of the UGT family-1 such as UGT72B27, there is no evidence of a metal ion bound in the GT-B structures [49]. Furthermore, the assay was utilized to establish the optimal conditions for the enzyme. As a result, with the UDP-Glo GT activity assay it was determined that the optimal conditions for the working enzyme, UGT72B27, are pH of 7.5, for 10 min, and at 30 °C, which is in accordance to the results obtained with the pH-sensitive assay for UGT72B27 [35].

**Figure 2:**
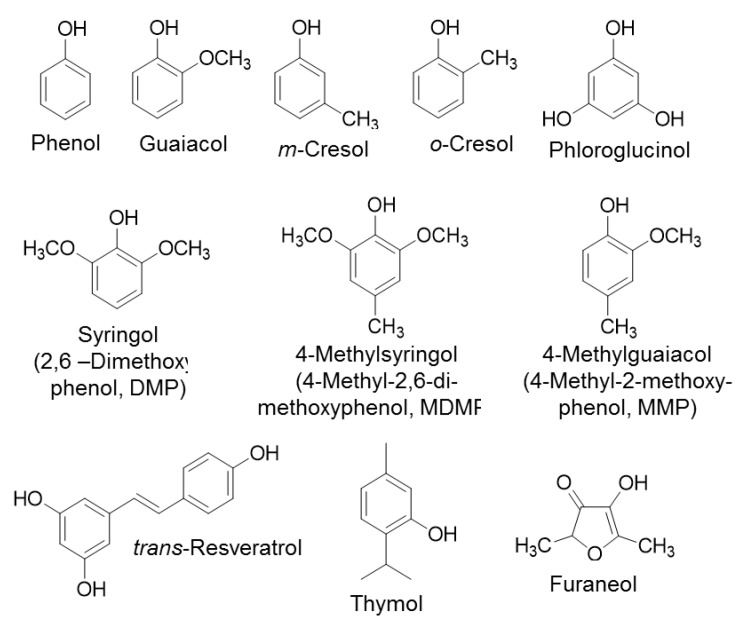
Chemical structures of substrates that were utilized in the detection of kinetic properties of UGT72B27.

Furthermore, the assay was tested and its functionality was verified by utilizing UGT72B27 with various naturally occurring substrates including guaiacol (2-methoxyphenol), resveratrol, thymol, syringol (2,6–dimethoxyphenol, DMP), 4-methylguaiacol (4-methyl-2-methoxyphenol, MMP), m-cresol, o-cresol, phloroglucinol, phenol, 4-methylsyringol (4-methyl-2,6-dimethoxyphenol, MDMP), and furaneol (Figure 2). The reaction was allowed to proceed at the optimal conditions and upon heat inactivation and addition of UDR the free UDP was converted to ATP generating light in a luciferase reaction (Figure 1). The intensity of the light was detected and was converted to the amount of UDP in µM using the previously obtained standard curve. The kinetic properties were calculated through the Michaelis-Menten equation. When comparing the results of the pH-sensitive assay determined by single measurements [35] with the results obtained with the UDP-Glo activity assay in 384-well plate, it can be seen that the two assays with the same GT and substrates yield different kinetic parameters (Table 2). The pH-sensitive assay showed higher K_M_ and k_cat_ values than the UDP-Glo^TM^ assay. In general, the pH-sensitive assay yielded higher catalytic activity (k_cat_/K_M_) values in comparison to the UDP-Glo assay (Table 2).

### 2.3. Phosphate Glycosyltransferase Activity Assay

The protocol was employed and adapted from the manufacturer. According to the manufacturer’s directions, the coupling phosphatase enzyme (CP) is to be added at the same time as the GT enzyme in a one-step reaction. Also, in this assay the GT reaction could not be stopped by the reagent provided by the manufacturer. We assume that this may be due to the fact that plant family-1 UGTs are metal-independent enzymes [49] (Figure S4). Therefore, the original protocol was tailored to UGT72B27 from *V. vinifera* and was executed in a two-step procedure. Following the GT reaction, the enzyme was inactivated by heating and then CP enzyme was added that the inorganic phosphate could be cleaved off the free UDP. The amount of the released phosphate was determined by malachite green reagents and the optical density was measured.

Prior to utilizing this assay, all the metrics and measurement thresholds were identified and tested to ensure it is able to function with plant GT’s and hydrophobic substrates. For example, protein purification protocols were adjusted to ensure all contents remain phosphate-free whilst conserving the proteins activity. Moreover, the CP concentration diluted in the 1× assay buffer was tested to ensure proper amounts and its feasibility and activity with the new assay conditions. Finally, the standard curve for phosphate was established. Furthermore, the assay was utilized to establish the optimal conditions for the enzyme, ensuring that the results obtained will be comparable to the already known optimal conditions. As a result, with the phosphate glycosyltransferase activity kit it was determined that the optimal conditions for the working enzyme, UGT72B27, are pH of 7.5, reaction time of 10 min, and at 30 °C, which is identical to the condition obtained by the UDP-Glo^TM^ assay.

**Figure 3:**
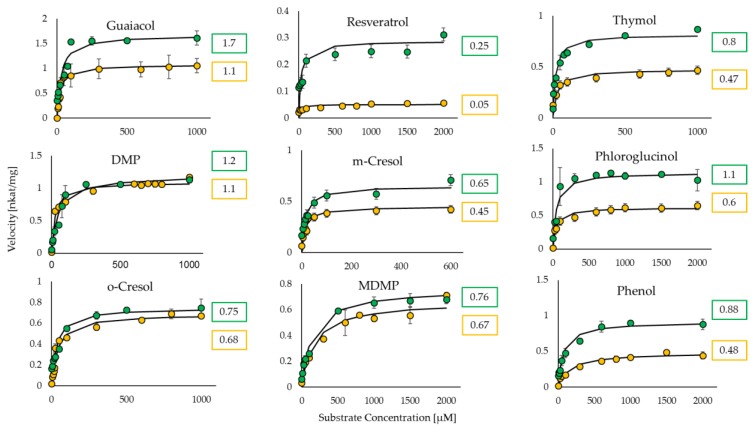
Graphical representation of the Michaelis–Menten curves for the substrates glycosylated by UGT72B27 quantified with UDP-Glo^TM^ assay (yellow) and phosphate GT activity assay (green). The numerical symbols represent the maximum velocity (V_max_) for each corresponding curve with the unit nkat/mg. *n* = 3.

Furthermore, the assay was tested and its functionality was verified by utilizing UGT72B27 with various naturally occurring phenolic substrates (Figure 2). The two-step reaction with the phosphatase enzyme was carried out and the OD_620_ was measured and converted to the phosphate concentration (pmol/well). With the obtained data, the kinetic properties were calculated through the Michaelis-Menten equation. Similar to the UDP-Glo^TM^ assay, the Phosphate GT assay showed lower K_M_ and k_cat_ values than the pH-sensitive assay. The kinetic values obtained via the Phosphate Activity Assay are very similar and not statistically different to those obtained through the UDP-Glo Activity Assay. The two assays, even though performed independently of each other, yielded similar kinetic results (Table 2; Figure 3). Meanwhile, the pH-sensitive assay yielded completely dissimilar kinetic values for the phenolic substrates (Table 2). The phenols used as acceptor substrates in this study act as weak organic acids, can release protons and therefore pretend a higher glycosyltransferase activity. This indicates that the pH-sensitive assay is unreliable in conducting kinetic analyses for UGTs with phenolic acceptors. 

### 2.4. TR-FRET UDP^2^ Transcreener Assay

The protocol was employed and adapted from the manufacturer. Prior to utilizing this assay, all the metrics and measurement thresholds were identified and tested to ensure it is able to function with plant GTs and hydrophobic substrates. For example, the Z’ value is a factor used to assess the quality of a screening assay. A Z’ value of 1 is ideal, 0.5-1 is an excellent assay, and below 0.5 is marginal indicating the assay is not suitable for screening purposes [50]. The Z’ value obtained for the assay was above 0.7 and the standard curve was established. However, from day-to-day the standard curve values changed and alternated deeming the antibody to be rather unstable. Although the manufacturer indicated that several thaw-and-freeze cycles are acceptable, in this case it was not. The antibody was precipitating on multiple occasions and even when fresh kit reagents were ordered, the attempts were all unsuccessful. Moreover, when the parameters were somewhat established the substrate screens and kinetics were vastly different between biological and even technical replicates. The Stop and Detect Buffer C was utilized to stop the GT reaction in a one-step format; however, this was not possible using UGT72B72 from *V. vinifera*. Therefore, the assay was altered by adding an additional step to heat stop the reaction and successfully terminate the GT. Despite this, the assay could not yield stable and consistent results (Figure S5).

## 3. Discussion

Over the years GTs have attracted significant attention in terms of cosmeceuticals, nutraceuticals, and drug design. However, the lack of availability of suitable plant GTs, and/or the requisite substrates, and more so stable HTP methods of detection, restricts the further application of these enzymes. Therefore, given the vast application spectrum and importance of UGTs, a high-throughput assay amenable to metal-independent plant GTs is pivotal. In this study, a HTP stable method was designed, which will allow for the discovery of new plant family-1 GTs, substrates, as well as uncover new and emerging applications. The advantages and drawbacks are thoroughly outlined, and their feasibility is described.

The pH-sensitive assay is based on the detection of the proton, which is released as a byproduct of the GT reaction (Figure 1). The released protons subsequently change the pH of the whole reaction. This change in pH is quantitatively determined by the addition of the pH indicator phenol red, which changes its color from yellow to red with pH values from 6.8 to 8.2, respectively. The assay was employed from [25] and used for plant GTs by [35]. We utilized this assay in separate 10 mm quartz cuvettes conducting single measurements, thus not being considered HTP. In this study, it was attempted to adapt this method to HTP format by engaging automatic injections and rapid absorbance reading with 1 s between each sample. Although the kinetic results obtained by [35] were reproduced under non-HTP conditions, the results were unreliable and unstable when the exact same GT and substrates were subjected to the HTP version of this assay. The kinetic values were different between all technical and biological replicates, as they were either too high or too low. It was not possible to reproduce the same results with the HTP method as in the study of [35] probably because the pH-sensitive assay is highly susceptible to disturbances. Since minimal proton concentration changes must be determined when performing the assay, a low concentrated buffer system is applied, making the system vulnerable to the use of acceptor substrates that can release protons themselves. This can cause for disturbances and may explain the reason of the pH-assay kinetic data being significantly higher compared to UDP-Glo^TM^ and Phosphate GT Activity assay (Table 2). Furthermore, a sufficient equilibration of the solutions at the fast measuring frequencies in the HTP mode is not guaranteed. Nevertheless, the pH-sensitive assay has already been successfully applied in HTP format for the screening of GT saturation mutagenesis libraries [44]. In this case, the assay was used for the screening of mutant GTs with the same oligosaccharides using suitable controls on each plate. As the system was optimized for screening in a solution with a weak buffer capacity, the authors noted that it is difficult to accurately quantify the initial enzyme reaction rates due to the drop in absorbance as the GT is added to the reaction mixture. In addition, the absorption coefficient of the pH indicator interferes with the measurement as the reaction progresses [51]. Different adjustments have to be made including the use of higher buffer concentration, larger amount of the indicator dye and increase in substrate concentration before the assay can be used for specific activity measurements [44]. However, increased substrate concentrations may limit the measurement of enzyme activity. In addition, chromophoric/fluorophoric substrates, such as in our case, also lead to erroneous results similar to substrates with acidic hydrogens (e.g., acids and phenolics) whereby the capacity of the weak buffer can be exceeded. All reasons above may contribute and explain the instability of the pH-sensitive assay when phenols are tested as substrates of GT in HTP format.

The pH-sensitive assay is undeniably cheap (0.01 € per assay), requires no specialized equipment, and can be used with crude protein extracts (Table 3). However, the drawbacks include its instability, unreliability across various substrates, and most importantly, it is unsuitable for HTP processing when different substrates should be tested. As the cost per assay is relatively low (Table 3), it can be utilized for an initial screen of substrates but is not feasible for quantitative experiments.

The UDP-Glo assay is based on the detection of the free UDP molecule, which results from the glycosyl transfer from the sugar donor to a sugar acceptor catalyzed by a GT enzyme. In a one-step addition, the free UDP is subsequently converted to ATP and luminescence is detected. The kinetics of the plant UGT72B27 along with the substrates from [35] were carried out and tested with the UDP-Glo^TM^ method. The assay allowed for the execution of multiple reactions providing a fast and easy readout. Overall, the mix and read format was straightforward and was easily applied to UGT72B27 with the corresponding substrates. According to the manufacturer’s instructions, the UDR is to be added directly to the GT reaction upon completion as it is meant to terminate the reaction and ultimately convert the UDP to ATP. However, following some test experiments it was noted that the plant GT reactions are not easily terminated by the UDR solution. When heat-inactivation was utilized to terminate the reaction, the UDP amounts yielded were approximately half the amount that were observed when the reaction was not terminated and UDP was added directly (Figure S3). We assume that the lack of the metal center of the plant family-1 GT prevents the ability to utilize the stopping agents of the kit to terminate the reaction. Therefore, when the reaction is not terminated and the UDR is directly added the GT is not completely stopped and it continues to function resulting in a higher amount of glycoside and detected UDP. Consequently, this method was tailored with an additional heat inactivation step to suit family-1 plant GTs. In one of our studies different UGTs from *Nicotiana benthamiana* were tested with different substrates under these tailored conditions and the results obtained with the tailored UDP-Glo^TM^ method were consistent and viable [38]. UDP-Glo^TM^ was also successfully applied for the rapid screening of sugar-nucleotide donor specificities of glycosyltransferases by determining their UDP-sugar hydrolase activities (Table 1) [45]. Therefore, in addition to the transferase activity of GTs, the innate UDP-sugar hydrolase activity of GTs can also be determined using the UDP-Glo^TM^ assay as it could not be detected by already existing LC-MS methods and methods using radiolabeled substrates. However, this UDP-sugar hydrolase activity must be taken into account when evaluating the UDP-Glo^TM^ assay results of alcohol acceptor substrates.

The UDP-Glo^TM^ assay is easy to handle, has a stable luminescence signal, and is sensitive (Table 3). This assay is three orders of magnitude more sensitive than the phosphate assay. Therefore, less enzyme is required to generate enough UDP above LOD to detect a significant signal compared with the phosphate assay. The drawbacks include the concentration of DMSO, which must be less than 10% otherwise it inhibits the luciferase enzyme and hinders the luminescence signal. Side innate activities such as UDP-glucose hydrolase could interfere with the results. The cost per assay is reasonable at 0.25 € per assay (Table 3) and can be employed in multiple screenings of substrates. In our hands, the UDP-Glo^TM^ has proven to be a stable and reliable HTP method for plant UGTs.

The Phosphate colorimetric assay is based on the detection of inorganic phosphate cleaved off by a phosphatase from the free UDP, which is the byproduct of the GT reaction (Figure 1). According to the manufacturer’s directions, the coupling phosphatase enzyme (CP) is to be added at the same time as the glycosyltransferase enzyme in a one-step reaction. The stopping agent provided by the kit interacts with the GT’s metal center and quenches its activity. The GTs previously utilized with were from different GT families (Table 1). When the plant GT with one of the substrates was carried out according to manufacturer’s conditions, the data did not yield quantifiable results as the GT reaction was not stopped with the provided buffer (Figure S4). Therefore, a tailored two-step phosphate colorimetric assay including a heat inactivation step was used to carry out the kinetics of UGT72B27 along with phenolic substrates (Table 2). The Phosphate Assay and the UDP-Glo^TM^ assay yielded nearly identical or similar Michaelis-Menten values (Figure 3) in contrast to the pH-sensitive assay, which yielded higher values due to the drawbacks discussed above.

The Phosphate assay is easy to handle, stable, and fast (Table 3). However, the one-step reaction suggested by the manufacturer was not applicable to the plant GT used in this study, but the developed two-step approach is nonetheless HTP and was successful. An important aspect is that no components of the reactions, including the purification of the protein, must contain any trace of phosphate as the malachite reagents are very sensitive and can interfere with the absorbance signal. Another drawback as with the UDP-Glo assay is that the concentration of solvents and detergents such as DMSO should not exceed 10% and they interfere with the malachite reagents and diminish the absorbance signal. The price per assay is estimated at 0.67 €, which is higher than the other assays described but is nonetheless reasonable. Overall, the phosphate assay is stable and can be easily utilized with plant UGTs in a two-step manner.

The transcreener UDP^2^ TR-FRET assay is based on the detection of the free UDP, which results from the glycosyl transfer from the sugar donor to a sugar acceptor catalyzed by a GT (Figure 1). It is a competitive immunoassay for UDP with a far-red, TR-FRET readout and has been used for discovery of GT inhibitors [41]. The Stop and Detect Buffer C is utilized to stop the GT reaction in a one-step format but did not work for the GT used in this study. Therefore, the assay was altered by adding an additional step to heat stop the reaction and successfully terminate the family-1 GT. The assay was validated by screening with 8000 compounds using a polypeptide N-acetylgalactosaminyltransferase containing a metal center as the target [41] but was not utilized with plant metal-independent GTs until now (Table 1). The attempts in this study at utilizing this assay with a family-1 plant GT were unsuccessful (Figure S5). Even when attempting to heat stop the enzyme and utilize the antibody conjugated with a fluorophore, the results were rather unstable and yielded day-to-day inconsistencies. Moreover, this assay was employed with other class of enzymes such as protein kinases and several considerations and drawbacks are present. For example, the interference by fluorescent compounds [52] or the inner-filter effect [53] need to be considered when employing such methods while utilizing red-shifted fluorophores for detection can reduce interferences caused by low-molecular weight compounds [52]. Furthermore, a drawback for this type of system is that a calibration of the antibody concentration based on the ATP/ADP is required to omit the variability of ATP [54]. In our experiments, the assay did not provide stable and consistent results when UGT72B27 from *V. vinifera* was used as UDP forming enzyme.

It is known that UGT72B27 prefers phenols as acceptor substrates including the smoke-derived phenolic xenobiotics guaiacol, MMP, DMP, MDMP, m-cresol, and o-cresol as well as furaneol (Figure 2) [35]. Kinetics of all of these phenolic substrates were successfully quantified with UDP-Glo^TM^ and Phosphate assay except for MMP and furaneol with the Phosphate assay (Table 2). Although, the structures of MMP, DMP, and MDMP are quite similar (Figure 2) the Phosphate assay did not provide reproducible data for MMP. However, LC-MS results confirmed the production of MMP glucoside [35] and UDP-Glo assay showed substantial kinetic data (Figure 3). Similarly, furaneol was unquantifiable with the Phosphate assay. Moreover, resveratrol yielded low kinetic values may be due to its significant hydrophobic properties and the need to dissolve it in DMSO amounts that exceed the UDP-Glo or Phosphate assays threshold. Thus, the high substrate concentrations measured with the corresponding assays yield poor results due to this interference. Moreover, the two assays were utilized in an initial substrate library screen to identify new substrates (Figure S1). The UDP-Glo^TM^ and Phosphate GT Activity assay have shown consistent and similar substrate specificity of UGT72B27 with 28 out of 32 new substrates (87.5%). Although, 4 substrates out of 32 (12.5%) showed dissimilar UDP amounts and thus GT activity when tested with the two assays. Therefore, it seems that there is no universal assay, which is suitable for all different substrates. Alternative HTP assays as well as LC-MS should be applied to avoid overlooking potential GT acceptors.

## 4. Materials and Methods

### 4.1. Materials and Chemicals

The working GT was prepared as previously described [35]. Chemicals were purchased with highest purity from Roth (Karlsruhe, Germany), Sigma-Aldrich (Steinheim, Germany), or Fluka (Steinheim, Germany) unless otherwise stated. The CLARIOstar plate reader (BMG Labtech, Ortenberg, Germany) was used for enzyme activity measurements. 

### 4.2. Heterologous Protein Expression with Phosphate-Containing Buffers

Protein expression was performed in the *E.coli* BL21 (DE3) pLysS cells harboring pGEX-4T-1 vector providing resistance against ampicillin and chloramphenicol and the *UGT72B27* sequence. A pre-culture was prepared by adding 2 µl of the cryostock culture to 10 mL lysogeny broth (LB) supplemented with 100 µl/mg ampicillin and 34 µl/mg chloramphenicol. The pre-culture was grown at 37 °C for 16 h in 50 mL Erlenmeyer flasks. The overnight culture was further propagated for 2–3 h in 400 mL of LB supplemented with ampicillin and chloramphenicol until the optical density (OD) at 600 nm reached 0.5–0.6. The expression of the protein of interest was induced by the addition of isopropyl-β-D-thiogalactopyranoside (IPTG) at a final concentration of 0.2 mM. The culture was grown for 20 h at 18 °C for 16 h shaking at 200 rpm. After harvesting the cells at 40,000 *g* (4 °C, 20 min), *E. coli* cell pellet was subjected to a freeze thaw cycle and resuspended in 2 mL of 2 mM Na-phosphate buffer (pH 8.0) and 10 µM of proteinase inhibitor, phenylmethylsulfonyl fluoride (PMSF). The cells were further disrupted via sonification (Sonopuls HD 2070 homogenizer) in phosphate-buffered saline (PBS) buffer for 6 cycles (30 s each cycle, 30 s pause, 10% power). After cell disruption, the cells were centrifuged at 200,000 *g* (4 °C, 20 min) and the clear supernatant containing the protein was obtained. Commercial GST-resin (Novagen, Darmstadt, Germany) was utilized and the recombinant GST-bound proteins were purified according to manufacturer’s instructions. The protein concentration was further determined via Roti-Nanoquant (Roth, Karlsruhe, Germany) according to manufacturer’s protocol and the presence of the recombinant proteins was verified by SDS-PAGE and Coomassie Staining (Figure S2).

### 4.3. Heterologous Protein Expression with Phosphate-Free Buffers

In order to subject the purified protein with the Phosphate GT assay, all buffers and components along the purification process must be phosphate-free. The protocol and all the concentration of all buffers remained the same as described above with the sole difference that the ‘phosphate’ content was replaced with ‘tris’. As an example, the PBS buffer was exchanged for the Tris-buffered saline (TBS) buffer. The purification of the protein was successful as determined by SDS-PAGE gels and Coomassie Staining.

### 4.4. Glycosyltransferase Activity Assays

The enzymatic reactions were performed with 50 mM Tris HCl (pH 7.5), 100 µM UDP-glucose, varying concentration of substrate (dissolved in DMSO), and 5 µg of purified UGT72B27 with a total reaction volume of 50 µL. All of the enzymatic reactions were executed in Eppendorf tubes utilizing incubators at 30 °C, 10 min, and shaking at 400 rpm. The kinetics and activity of the GT was analyzed via various activity assays described below.

### 4.5. Determination of Kinetic Parameters Using a pH-Sensitive Colorimetric Assay

A calibration curve was established as previously described [25]. In brief, the calibration curve was arranged in a 2 mM sodium phosphate buffer (pH 8, 120 µL) containing 0.01 mM phenol red, 0.1 mM MnCl_2_, 10 mM substrate, and 5 µg purified UGT72B27, with the addition of varying amounts of 10 mM hydrochloric acid to a range of final concentration of 0.1, 0.2, 0.3, 0.4, 0.5, and 0.6 mM. The respective OD_557_ was recorded. A quantitative linear relationship between proton concentration and absorbance was established.

For the determination of kinetic parameters, 0.01 mM phenol red, 0.1 mM MnCl_2_, varying substrate concentration from 0-2000 µM, and 5 µg purified UGT72B27 were mixed with phosphate buffer (2 mM, pH 8). The assay was commenced by the addition of the UDP-glucose to a final concentration of 2 mM and a final reaction volume of 120 µL. The respective absorbance at 557 nm was recorded for each sample at 10 sec intervals for a total of 3 min until a constant signal was obtained. The enzyme activities were calculated from the calibration curve. All measurements were executed in triplicates and the values were averaged. Finally, K_M_ and V_max_ were calculated by nonlinear regression of the Michaelis-Menten equation using the Microsoft Excel Solver.

In establishing a high-throughput process for the pH-sensitive colorimetric assay, the reaction conditions remained the same as previously described. The enzymatic reaction was prepared in 96-well plate in a total volume of 120 µL. The reaction was commenced with the addition of UDP-glucose utilizing the injector function of the multi-plate reader. The components were thoroughly ‘shaken’ and incubation took place inside the plate reader at the optimal temperature. Upon the particular reaction time the OD was directly measured. All measurements were executed in triplicates with negative and positive controls.

### 4.6. Determination of Kinetic Parameters Using the UDP-Glo Glycosyltransferase Assay

The commercial kit UDP-Glo Glycosyltransferase Assay was purchased from Promega (Mannheim, Germany). Parts of the assay were established according to manufacturer’s protocol meanwhile, other parts were tailored to fit the specificity of the working GT. The UDP-Detection Reagent (UDR) was prepared and the reaction was executed in 384-well plates. To estimate the amount of UDP produced in the enzymatic reaction, a UDP standard curve was established according to manufacturer’s conditions. Briefly, a 0-1000 µM UDP standard was prepared in a 384-well plate. A 1000 µM UDP solution was added in the first well and was serially diluted across 24 wells with the last well serving as a no-UDP control. Respectively, the UDR was added to the corresponding wells, the plate was incubated at room temperature for 60 min, and finally the relative luminescence (RLU) signal was measured with the CLARIOStar microplate reader. A calibration curve was extrapolated from the average of these measurements (Figure S6). Moreover, along with the UDP standard curve an enzyme titration was performed where 10 µg/µL of UGT72B27 was serially diluted and subjected to an incubation of one hour at 30 °C with acceptor (thymol) and donor substrate (UDP-glucose) consequently heat stopped to terminate the reaction (Figure S7). Furthermore, the limit of detection (LoD = limit of blank + 1.645 * standard deviation of a low concentration sample) was determined for UDP-Glo^TM^ to be 13604.18 RLU (7.4 nM UDP) according to [55], while the manual of the UDP-Glo kit states that the assay detects 0.1–0.5 pmol UDP with a more than twofold difference over the background. The different numbers are due to the different calculation methods. The manufacturer also suggests using a commercially available Ultra-pure UDP, which could also contribute to the different values.

For the determination of kinetic parameters, a 50-µL GT reaction was prepared as follows: 50 mM Tris/HCl (pH 7.5), 100 µM UDP-glucose, 5 µg purified UGT72B27, with varying concentrations of substrate ranging from 0–2000 µM. The reaction was commenced by the addition of the sugar-donor, UDP-glucose. Termination of the enzymatic reaction was executed by incubating at 75 °C for 10 min and subsequently addition of 5 µL of UDR to 5 µL of the enzymatic reaction in 384-well plate (1:1 ratio). The plate was allowed to equilibrate at room temperature for 60 min in the dark. Following the incubation period, the relative luminescence (RLU) signal was measured via the plate reader. The enzyme activities were calculated from the calibration curve and the respective RLU signal. All measurements were executed in triplicates with appropriate controls and the values were averaged.

### 4.7. Determination of Kinetic Parameters Using the Phosphate Glycosyltransferase Activity Kit

The commercial kit Phosphate Glycosyltransferase Activity kit was purchased from R&D Systems (Abingdon, UK). The principle of the assay along with the materials provided were utilized according to manufacturer’s conditions. The method was tailored to fit the needs and specificity of the working GT. To estimate the amount of UDP produced in the enzymatic reaction, a phosphate standard curve was established according to manufacturer’s conditions. Briefly, 100 µM of the phosphate standard was prepared in 1× assay buffer (500 µL of phosphatase buffer 1, 500 µL of 100 mM MnCl_2_, 4.0 mL of deionized water). The standard was serially diluted across 12 wells in a 96-well plate. To each well, 30 µL of malachite green reagent A and B were added according to manufacturer’s protocol. Subsequently, the plate was incubated for 20 min at room temperature and the optical density (OD) at 620 nm was measured using the CLARIOstar microplate reader. Each dilution was performed in triplicates and the average was utilized to produce a calibration curve with optical density at 620nm versus concentration of UDP (Figure S6). Moreover, along with the UDP standard curve an enzyme titration was performed where 10 µg/µL of UGT72B27 was serially diluted and subjected to an incubation of one hour at 30 °C with acceptor (thymol) and donor substrate (UDP-glucose) consequently heat stopped to terminate the reaction (Supplementary Figure S7). Furthermore, the limit of detection (LoD) was determined for Phosphate GT Activity assay to be 0.182 OD_620_ (6.9 µM UDP) according to [55]. The manual of the phosphate kit, which is based on the measurement of the chromogenic complex formed by malachite green, ammonium molybdate and phosphate states that phosphate concentrations between 1 μM and 1 mM, with a lower limit of detection of approximately 0.1 nmol, can be directly determined, which equals 4.3 µM, 4.3 mM, and 0.43 nmol UDP, respectively. Again, the different numbers are probably due to the different calculation methods.

For the determination of kinetic parameters, a 50-µL GT reaction was prepared as follows: 200 mM Tris/HCl (pH 7.5), 100 µM UDP-glucose, 5 µg purified UGT72B27, with varying concentrations of substrate ranging from 0–2000 µM. The reaction was commenced by the addition of the sugar-donor, UDP-glucose. Termination of the enzyme reaction was executed via heating the reaction to 75 °C for 10 min. Subsequently, the reaction was centrifuged for 20 min to remove any unbound substrate and 25 µL was aliquoted into a 96-well plate. Correspondingly, 20 µL of 1× assay buffer and 5 µL of 20 µg/µL coupling phosphatase was added. Together, all components in the microplate were incubated at 37 °C for 60 min. Following the 2-step incubation, 30 µL of malachite green reagent A, 100 µL of distilled water, and 30 µL of malachite green reagent B were added to the corresponding well containing the GT reaction in a step-wise manner. Following a 20 min room temperature incubation, the OD at 620 nm was measured using CLARIOstar. The amount of phosphate detected was directly proportional to the amount of UDP produced. The calibration curve was utilized to determine the enzymatic activity. All measurements were executed in triplicates with appropriate controls and the values were averaged.

### 4.8. Determination of Kinetic Parameters Using the TR-FRET UDP^2^ Transcreener Assay

The commercial kit Transcreener UDP^2^ Glycosyltransferase assay was purchased from BellBrook Labs (Wisconsin, USA). The principle of the assay along with the materials provided were utilized according to manufacturer’s conditions. Before commencing the measurements, the optimization of the maximum TR-FRET Window of the plate reader and the determination of the optimal UDP HiLyte647 Tracer concentration were conducted according to manufacturer’s conditions. To estimate the amount of UDP produced in the enzymatic reaction, a UDP standard curve was established according to manufacturer’s conditions. Briefly, a 12-point standard curve was prepared using concentrations of UDP-glucose and UDP from 1 µM to 1000 µM. Fifteen µL of each standard was aliquoted into the corresponding well of a 384-well plate and 5 µL of the UDP-detection mixture (8 nM UDP^2^ anitbody-Tb, 1× Stop and Detect Buffer C, 4 × (EC_85_) UDP HiLyte647 Tracer) was added. The plate was incubated for 60 min at room temperature and the TR-FRET signal was measured via the plate reader. Each dilution was performed in triplicates and the average was utilized to produce a calibration curve with UDP (µM) versus TR-FRET 665:615 ratio.

For the determination of kinetic parameters, a 50 µL GT reaction was prepared as follows: 200 mM Tris/HCl (pH 7.5), 100 µM UDP-glucose, 5 µg purified UGT72B27, with varying concentrations of substrate ranging from 0–2000 µM. The reaction was commenced by the addition of the sugar-donor, UDP-glucose. Termination of the enzyme was executed via heating the reaction to 75 °C for 10 min. Subsequently, the reaction was centrifuged for 20 min to remove residues of unbound substrate and 15 µL of the enzymatic reaction was aliquoted into a 384-well plate. Five µL of the 1X UDP Detection Mixture was added to each corresponding well and the plate was incubated for 60 min at room temperature. The TR-FRET signal was detected via plate reader and the UDP amounts were extrapolated from the standard curve. The TR-FRET signal is inversely proportional to the amount of UDP present in the reaction. All measurements were executed in triplicates with appropriate positive and negative controls.

## 5. Conclusions

This comparison has shed light on the applicability of different commercial and simple GT enzyme assays for HTP purposes. This study has proven that HTP quantification is possible, and as UGTs are promiscuous, more than one detection method is beneficial. Even with the tailored assays and the extra steps that are taken to ensure feasibility the procedures remain fast, simple, safe and easy to handle in comparison to LC-MS or use of radiolabeled sugars. Therefore, in this study two new methods have been established that could be utilized in screening and kinetic determination of various substrates and plant family-1 GTs. The UDP-Glo^TM^ and the phosphate GT activity assay have proven to be compatible and effective when experimenting with plant family-1 UGTs and phenolic substrates.

## Figures and Tables

**Table 1:** Application examples of the investigated assays in previous studies. ND, not determined; MD, metal-dependence; R, References.

Assay	Application	Enzyme	Species	GT Family	Fold	Mechanism	MD	R
pH-Sensitive	-Screening GTs -Saturation mutagenesis library	GTB	*H. sapiens*	6	GT-A	Retaining	Mn^2+^	[25]
		GTA	*H. sapiens*	6	GT-A	Retaining	Mn^2+^	[25]
		GalT1	*H. sapiens*	7	GT-A	Inverting	Mn^2+^	[44]
		LgtB	*N. meningitidis*	25	ND	Inverting	Mn^2+^	[44]
		HP0826	*H. pylori*	25	ND	Inverting	ND	[44]
Nucleotide- Glo^TM^ (UDP, GDP, UMP)	-Sugar-nucleotide donor specificity -Screening of GT inhibitors	POMGNT1	*H. sapiens*	13	GT-A	Inverting	Mn^2+^	[45]
		B4GAT1	*H. sapiens*	49	ND	Inverting	Mn^2+^	[45]
		SpGtfA (OGT)	*S. pneumonia*	41	GT-B	Inverting	ND	[36,45]
		DdAgtA	*D. discoideum*	77	ND	Retaining	Mn^2+^	[45]
		POGLUT1	*H. sapiens*	90	ND	Inverting	ND	[45]
		β4 Gal-T1	*Bos taurus*	7	GT-A	Inverting	Mn^2+^	[36,45]
		LARGE1	*H. sapiens*	49/8	ND/GT-A	Inverting/Retaining	Mn^2+^	[45]
		PglC	*C. jejuni*	4	GT-B	Retaining	Mn^2+^/Mg^2+^	[46]
		PglC	*H. pullorum*	4	GT-B	Retaining	Mn^2+^/Mg^2+^	[46]
		WecA	*T. maritima*	4	GT-B	Retaining	Mn^2+^/Mg^2+^	[46]
		UGT1A1	*H. sapiens*	1	GT-B	Inverting	ND	[36]
		GTB	*H. sapiens*	6	GT-A	Retaining	Mn^2+^	[36]
		GALNT1	*H. sapiens*	27	GT-A	Retaining	Mn^2+^	[36]
		ST6GAL1	*H. sapiens*	29	ND	Inverting	ND	[37]
		UGT2B17	*H. sapiens*	1	GT-B	Inverting	ND	[36]
		FUT2	*H. sapiens*	11	ND	Inverting	ND	[36]
		FUT3	*H. sapiens*	10	GT-B	Inverting	ND	[36]
		FUT7	*H. sapiens*	10	GT-B	Inverting	ND	[36]
		IRX10-L	*A. thaliana*	47	GT-B	Inverting	ND	[47]
		AtFUT1	*A. thaliana*	37	GT.B	Inverting	ND	[48]
PO_4_ GT Assay	-Kinetic analyses	TcdB	*C. difficile*	44	ND	Retaining	ND	[39]
		KTELC1	*H. sapiens*	90	ND	Inverting	ND	[39]
		ST6GAL1	*H. sapiens*	29	ND	Inverting	ND	[39]
UDP^2^ TR-FRET	-Discovery of GT inhibitors	GALNT3	*H. sapiens*	27	GT-A	Retaining	Mn^2+^	[41]

**Table 2:** Comparison of kinetic values of purified UGT72B27 enzyme obtained via three different detection methods. (**a**) pH-sensitive assay, data taken from [35], obtained by single measurements, (**b**) UDP-Glo^TM^ assay, measured in 384-well plate, and (**c**) phosphate glycosyltransferase assay, measured in 96-well plate. Substrate concentrations were varied from 10–3000 μM. Guaiacol: 2-Methoxyphenol, DMP: 2,6-Dimethoxyphenol, MMP: 2-Methoxy-4-methylphenol, MDMP: 4-Methyl-2,6-dimethoxyphenol. n = 3. (*) since the publication, this value was corrected.

Substrate	a. pH-Sensitive Assay			b. UDP-Glo^TM^ Assay			c. Phosphate GT Assay		
	K_M_ (µM)	k_cat_ (s^−1^)	k_cat_/K_M_ (mM^−1^ s^−1^)	K_M_ (µM)	k_cat_ (s^−1^)	k_cat_/K_M_ (mM^−1^ s^−1^)	K_M_ (µM)	k_cat_ (s^−1^)	k_cat_/K_M_ (mM^−1^ s^−1^)
Guaiacol	*32 ± 1	2.3 ± 0.02	72.5 ± 3	23 ± 1	0.08 ± 0.02	3.7 ± 1	28 ± 3	0.13 ± 0.003	4.7 ± 0.6
trans-Resveratrol	36 ± 5	0.6 ± 0.05	17.0 ± 3.8	21 ± 5	0.004 ± 0.0001	0.2 ± 0.05	15 ± 1	0.02 ± 0.002	1.3 ± 0.2
Thymol	53 ± 0.25	0.7 ± 0.07	13.5 ± 1.4	28 ± 7	0.04 ± 0.003	1.4 ± 0.1	20 ± 1	0.07 ± 0.001	3.3 ± 0.2
DMP	211 ± 52	1.9 ± 0.05	8.8 ± 2.4	23 ± 3	0.09 ± 0.003	3.7 ± 0.6	41 ± 4	0.1 ± 0.004	2.3 ± 0.3
MMP	115 ± 22	0.9 ± 0.01	8.0 ± 1.6	41 ± 9	0.06 ± 0.001	1.4 ± 0.3			
m-Cresol	48 ± 19	0.4 ± 0.02	7.9 ± 3.5	14 ± 3	0.04 ± 0.002	2.6 ± 0.6	15 ± 2	0.05 ± 0.0005	3.4 ± 0.5
Phloroglucinol	77 ± 10	0.5 ± 0.05	7.1 ± 1.6	35 ± 4	0.05 ± 0.006	1.3 ± 0.3	47 ± 4	0.09 ± 0.006	1.9 ± 0.3
o-Cresol	148 ± 14	0.5 ± 0.04	3.6 ± 0.6	40 ± 5	0.05 ± 0.002	1.4 ± 0.2	32 ± 2	0.06 ± 0.005	1.8 ± 0.3
MDMP	278 ± 22	0.5 ± 0.04	1.9 ± 0.3	173 ± 28	0.05 ± 0.005	0.3 ± 0.07	143 ± 13	0.06 ± 0.002	0.5 ± 0.1
Phenol	326 ± 83	0.6 ± 0.04	1.8 ± 0.6	153 ± 24	0.04 ± 0.002	0.3 ± 0.06	62 ± 0.5	0.07 ± 0.007	1.1 ± 0.1
Furaneol	478 ± 45	0.5 ± 0.04	1.0 ± 0.2	453 ± 35	0.02 ± 0.002	0.04 ± 0.007			

**Table 3:** Advantages and disadvantages of the three different detection methods—the pH-sensitive assay, UDP-Glo^TM^ assay, and phosphate glycosyltransferase assay.

	pH Sensitive Assay	UDP-Glo Assay	Phosphate GT Activity Assay
**Advantages**	-Simple, cheap, and fast -Requires no specialized equipment -Can be used with crude protein -Useful for screening of one acceptor with multiple GTs	-Simple, fast, and sensitive -Easy handling -High-throughput -Stable luminescence signal -Requires no labelled substrate -Most sensitive assay due to lowest LoD	-Simple, fast, and sensitive -Easy handling and detection -High-throughput -Stable absorbance signal -Requires no labelled substrate
**Disadvantages**	-Unstable -Not applicable for HTP analyses of kinetic data with phenolics	-UDP-glucose hydrolase activity must be taken into account -DMSO concentration must be under 10% -Adjustment necessary if reaction is not stopped by the provided reagent	-One-step reaction is not applicable if GT is not stopped by the provided reagent -UDP-glucose hydrolase activity must be taken into account -All components of the reaction must be phosphate-free -DMSO concentration must be under 10%

## Supplementary Materials

Supplementary materials can be found at https://www.mdpi.com/1422-0067/21/6/2208/s1.

## Abbreviations

GST	glutathione S-transferase
GTs	glycosyltransferases
HTP	high-throughput
LC-MS	liquid chromatography-mass spectrometry
TR-FRET	Time-Resolved Fluorescence Resonance Energy Transfer
UDP Glc	Uridine diphosphate glucose
UGT	Uridine diphosphate dependent glycosyltransferases
*V. vinifera*	*Vitis vinifera*
VvUGT	*V. vinifera UGT*
