# Supplementary material for: Comparative Analysis of High-Throughput Assays of Family-1 Plant Glycosyltransferases
Source: Int J Mol Sci. 2020 Mar 23;21(6):2208. doi: 10.3390/ijms21062208 (PMC7139940; doi:10.3390/ijms21062208)
Supplement: Supplementary file 1 [file ijms-21-02208-s001.pdf]

Supplementary Material

Comparative analysis of high-throughput assays of family-1 plant glycosyltransferases

Kate McGraphery [1], Wilfried Schwab [1,*]

[1] Biotechnology of Natural Products, Technische Universität München, 85354 Freising, Germany;

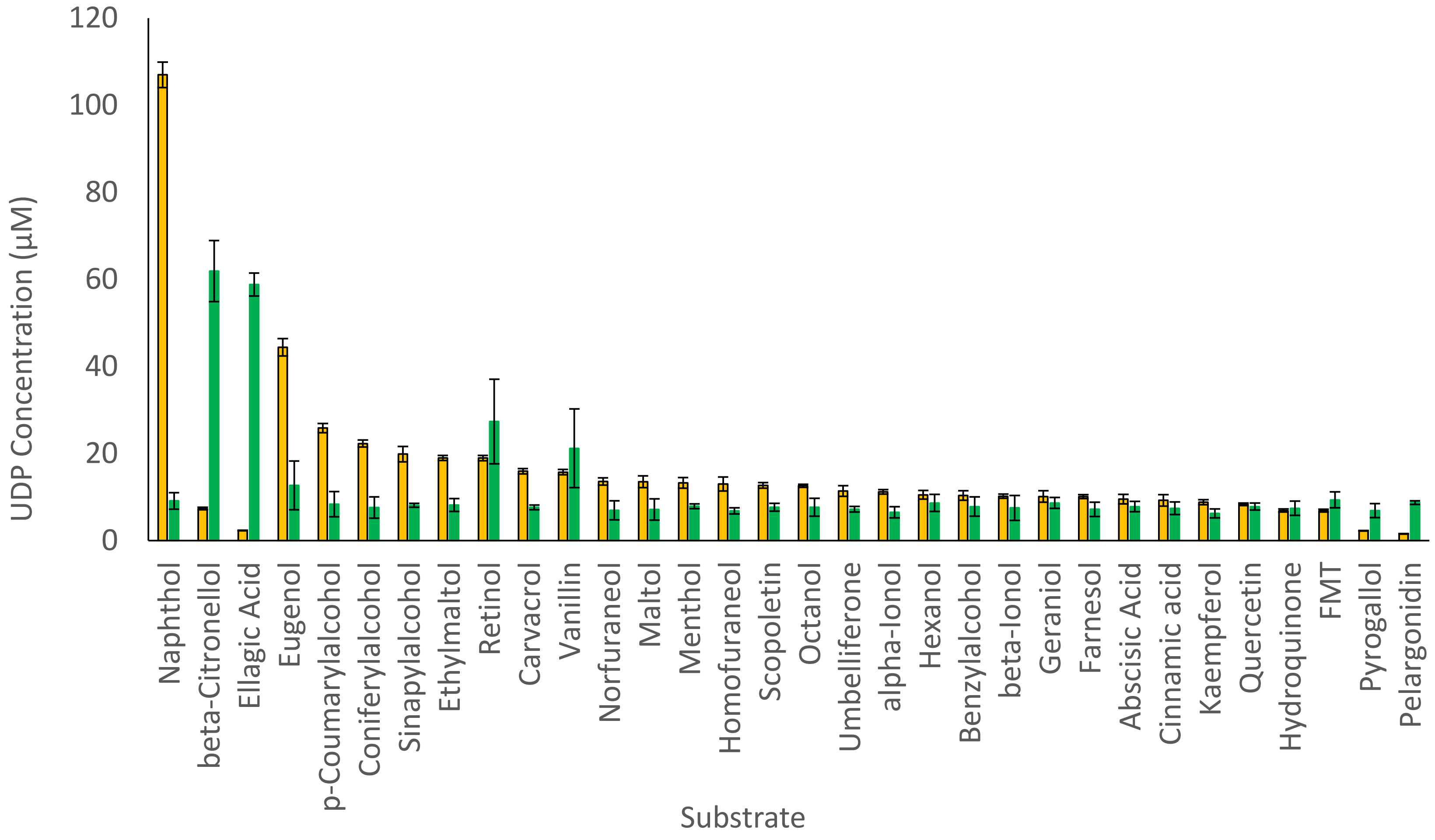

Figure S1: Comparison of the substrate screening of UGT72B27 with UDP-Glo (yellow) and Phosphate Assay (green). n=3.

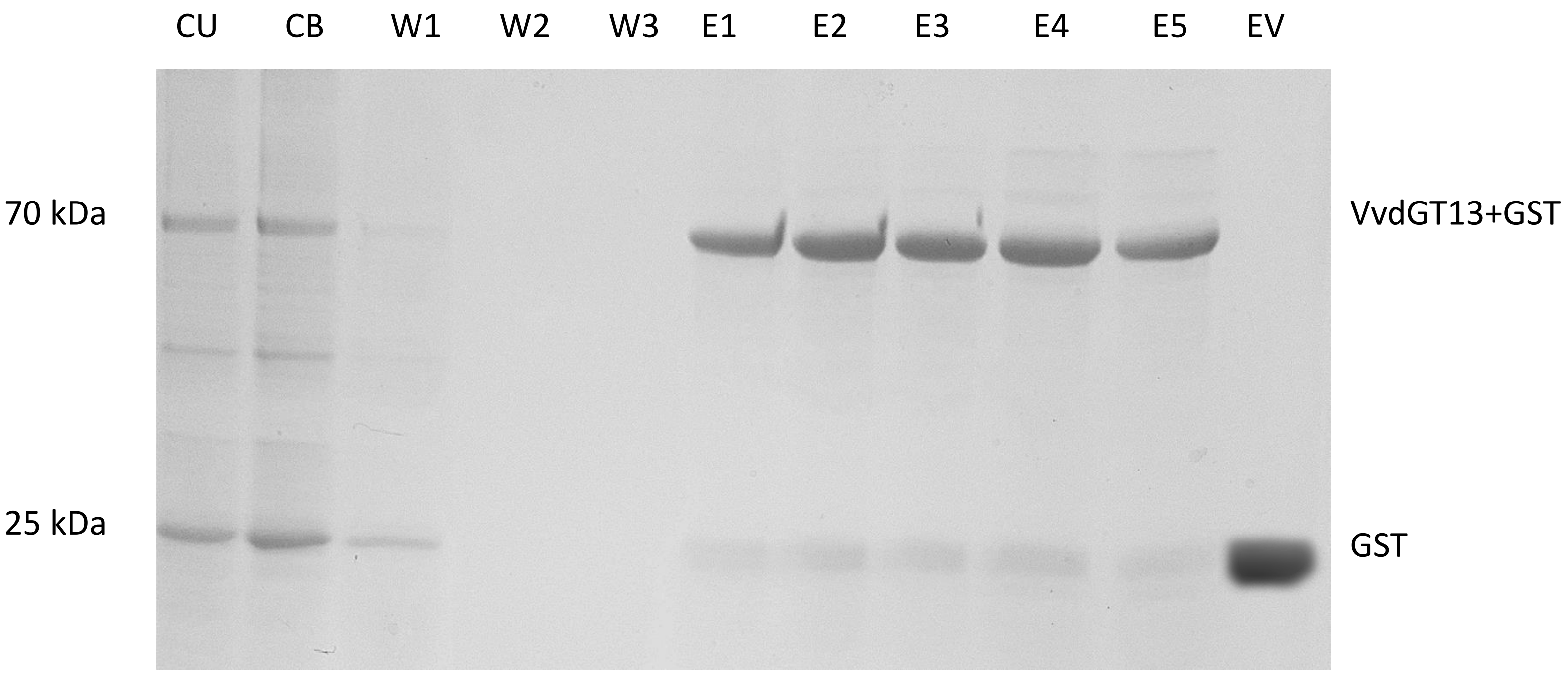

Figure S2: Image of the resulting Coomassie stained SDS-PAGE following the protein purification of the recombinant UGT72B27. CU = Crude protein unbound to GST resin. CB = Crude protein following the binding. W1 = first buffer wash. W2 = second buffer wash. W3 = third buffer wash. E1 – E5 = first to fifth elution of the purified recombinant protein. EV = empty PGEX-4T1 vector containing only the GST vector without the recombinant protein.

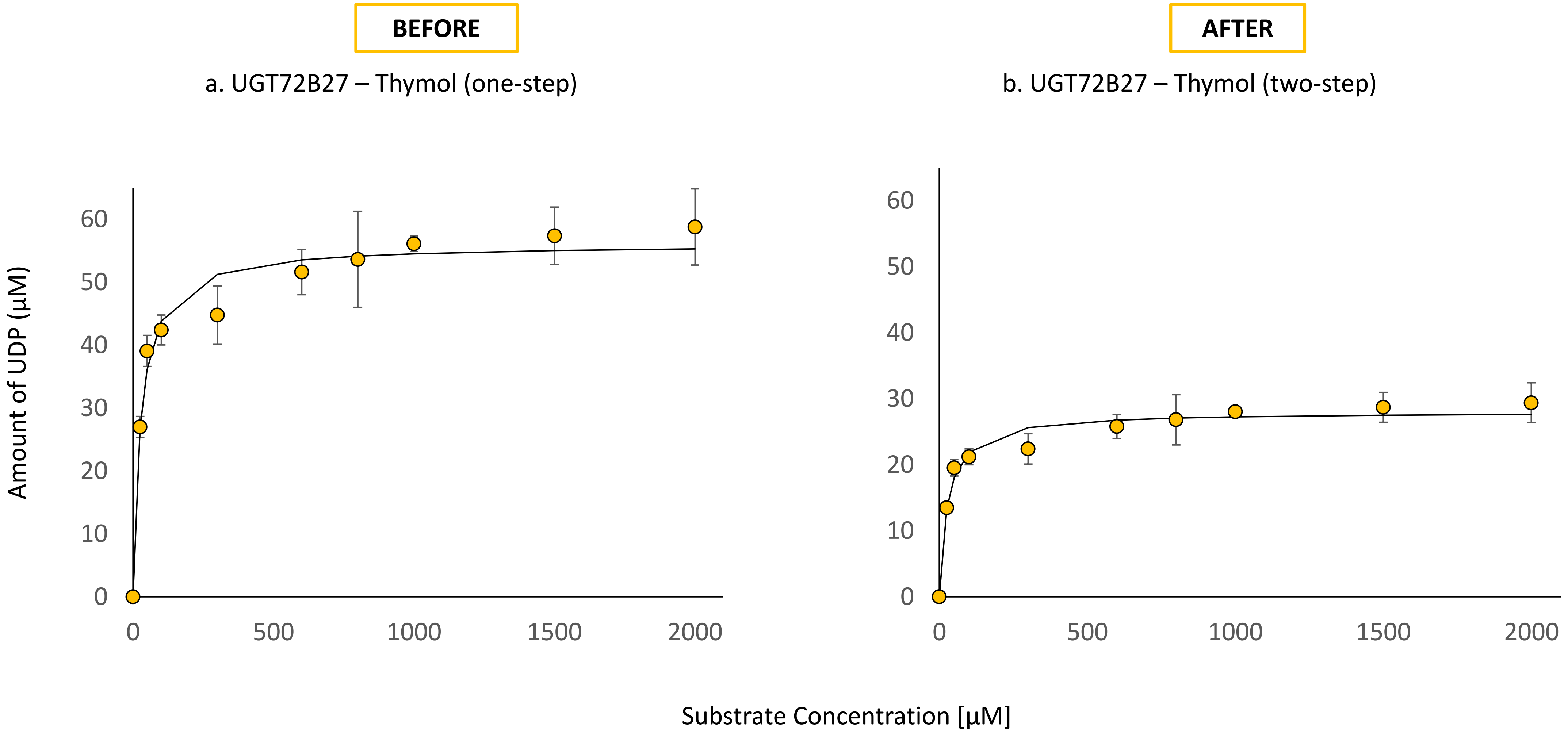

Figure S3: Graphical representation of the optimization of the UDP-Glo Assay, (a) UGT72B27 with thymol employed according to manufacturer‘s conditions in a one-step manner where kinetic data was calculated. (b) UGT72B27 with thymol executed in two-steps with an additional heat stop GT inactivation prior to UDR addition, ultimately tailored to fit plant GTs.

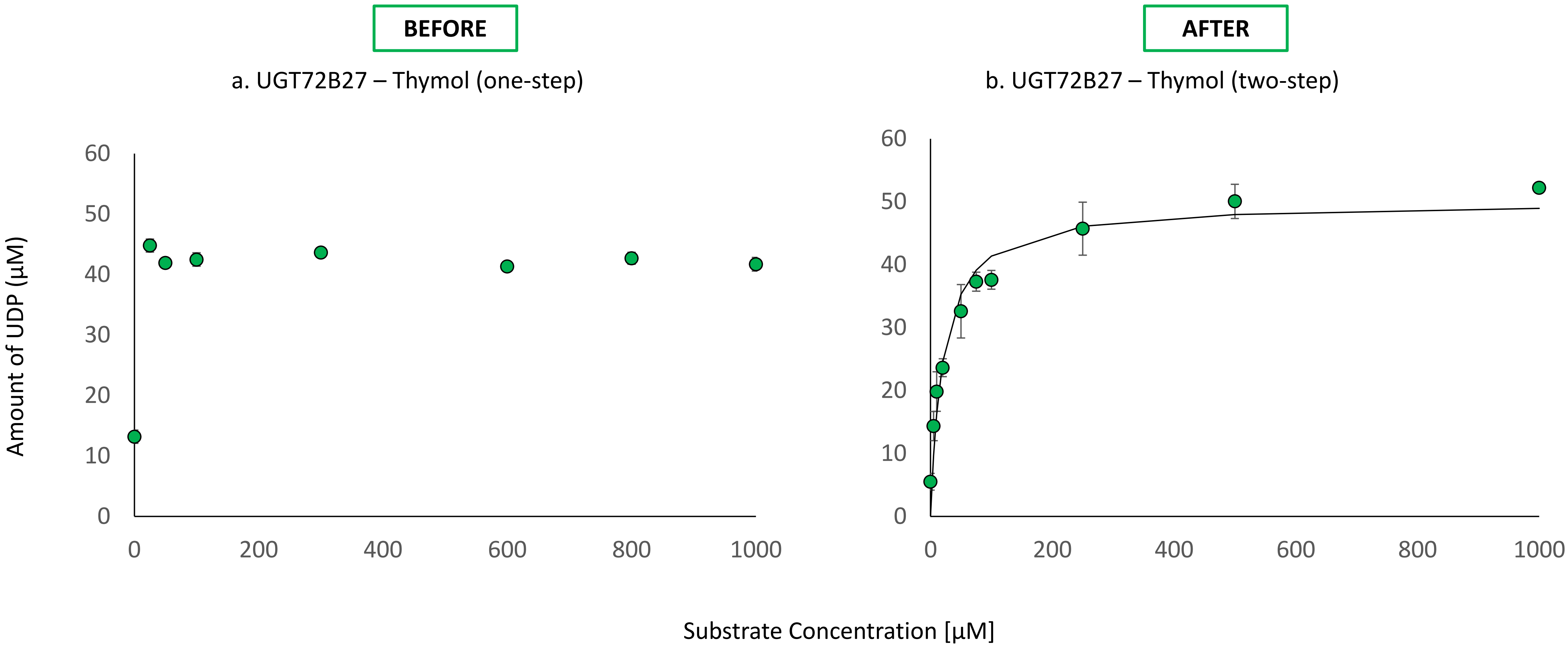

Figure S4: Graphical representation of the optimization of the Phosphate assay, (a) UGT72B27 with thymol employed according to manufacturer's conditions in a one-step manner where kinetic data could not be calculated. (b) UGT72B27 with thymol executed in two-steps with an additional heat stop GT inactivation prior to CP addition, ultimately tailored to fit plant GTs.

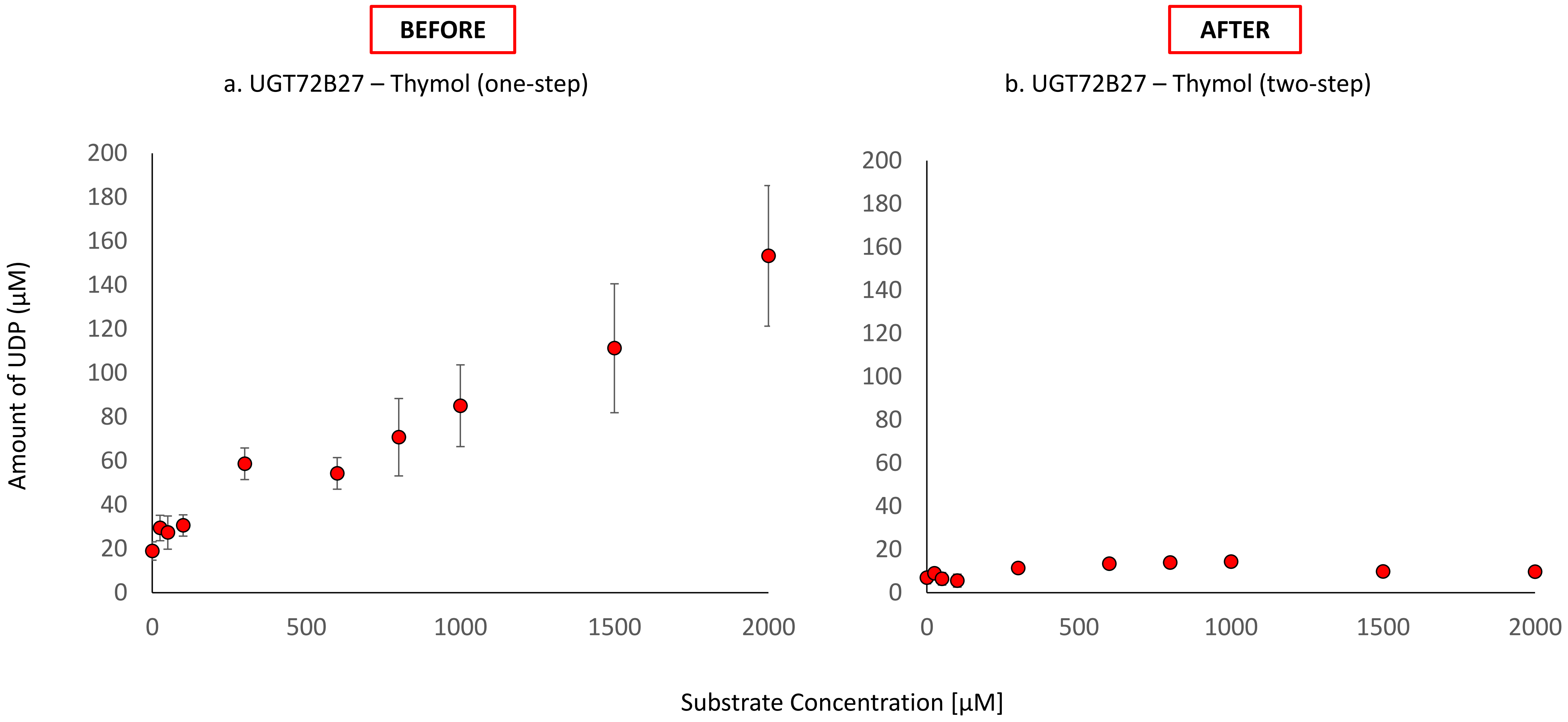

Figure S5: Graphical representation of the optimization of the UDP TR-FRET immunoassay, (a) UGT72B27 with thymol employed according to manufacturer‘s conditions in a one-step manner where kinetic data could not be calculated. (b) UGT72B27 with thymol executed in two-steps with an additional heat stop GT inactivation prior to antibody addition.

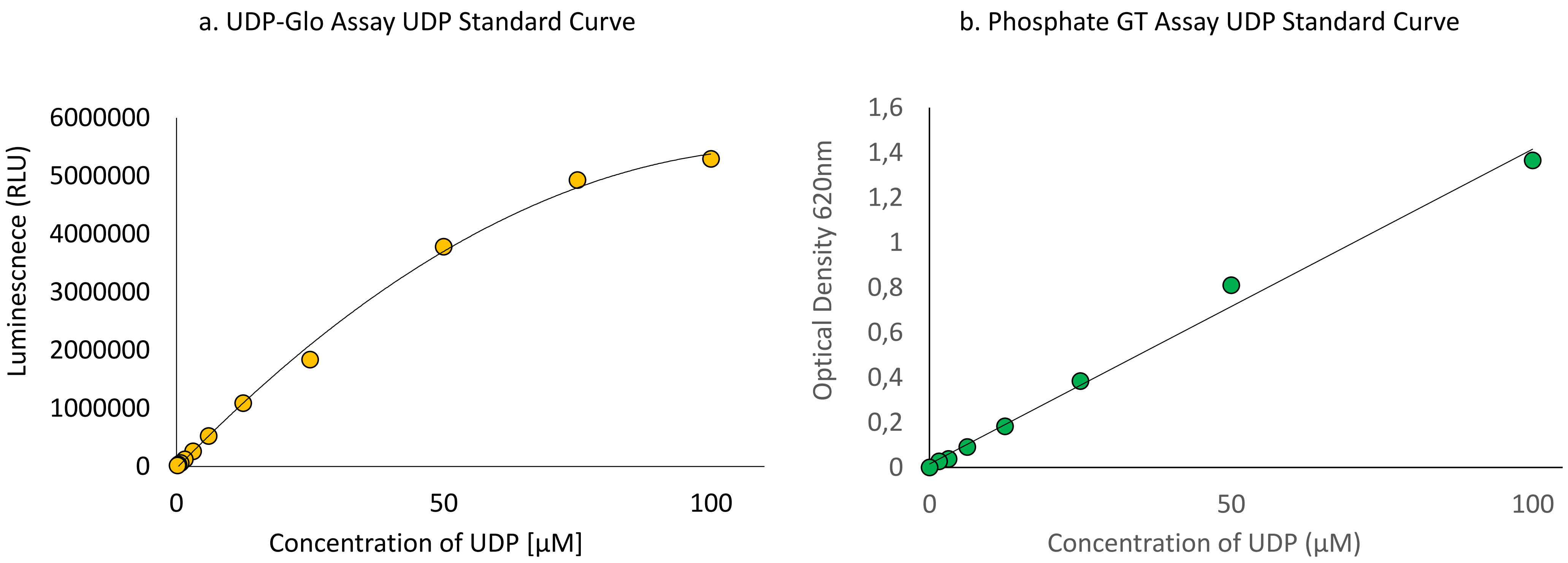

Figure S6: Standard Curves for UDP concentration were established with the (a) UDP Glo Assay and (b) Phosphate GT Assay. The luminescence and the optical density are directly proportional to the concentration of UDP produced in both assays. n=2.

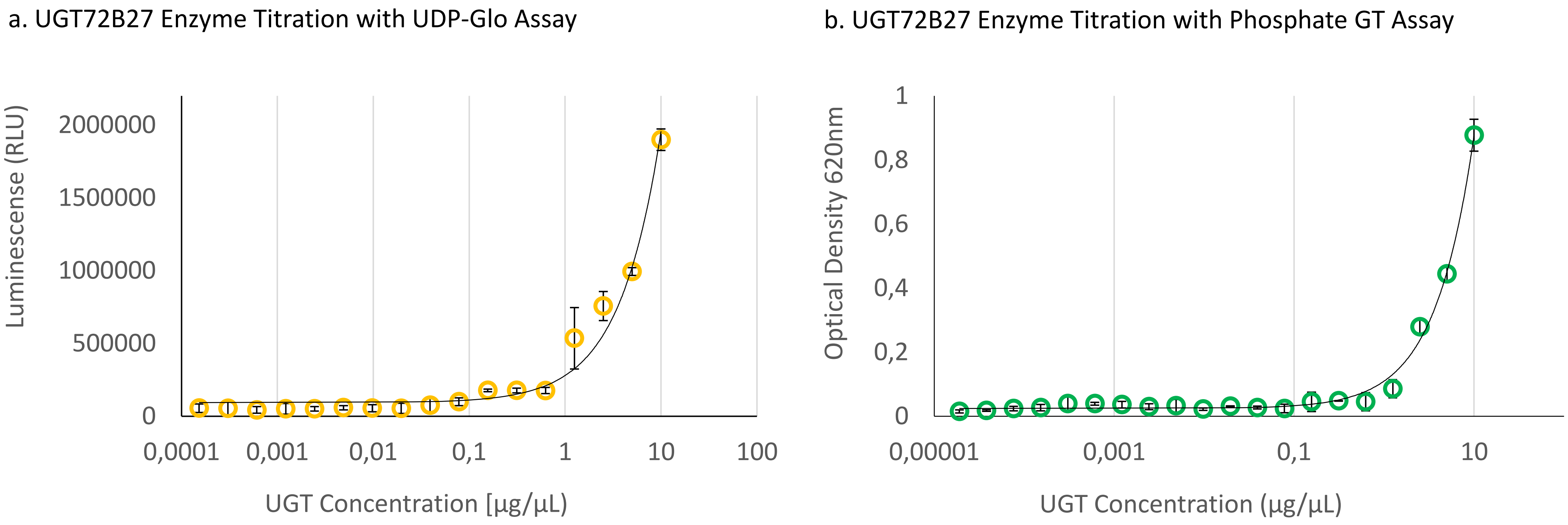

Figure S7: Detection of UGT72B27 activity with acceptor (thymol) and donor (UDP-glucose) substrates with (a) the UDP-Glo assay and (b) Phosphate GT activity assay. The enzyme titration was conducted according to manufacturer's protocol under optimal enzyme conditions along with the additional steps tailored to family 1 plant GTs. The enzyme is more active and best detectable by both assays at a range of concentrations from 1.25 to 10 µg/µL. n=2.
